# Amelogenesis Imperfecta with Nephrocalcinosis: A Rare Association in Siblings

**DOI:** 10.7759/cureus.5060

**Published:** 2019-07-01

**Authors:** Pramod Reddy, Swathi Aravelli, Soujanya Goud, Loka Malathi

**Affiliations:** 1 Conservative Dentistry and Endodontics, Saint Joseph Dental College, Eluru, IND; 2 Conservative Dentistry and Endodontics, Mallareddy Dental College for Women, Malkajgiri, IND; 3 Conservative Dentistry and Endodontics, Army College of Dental Sciences, Malkajgiri, IND; 4 Oral Medicine and Radiology, Sri Venkateshwara Dental Hospital, Adilabad, IND

**Keywords:** amelogenesis imperfecta, enamel hypoplasia, nephrocalcinosis, retained deciduous teeth, unerupted teeth

## Abstract

Enamel hypoplasia secondary to amelogenesis imperfect (AI) is one of the common developmental disturbances associated with the oral cavity. AI in association with multiple unerupted teeth is a rare entity, and in adolescence it not only has an affect on esthetics but also has an impact on the psychological status of the person. AI has been reported with other systemic anomalies previously. We report a case of AI in association with multiple unerupted teeth and nephrocalcinosis in siblings. The present case also highlights the importance of systemic examination and investigations in planning the treatment of a patient with AI.

## Introduction

Amelogenesis Imperfecta (AI) represents a group of developmental conditions, genomic in origin, which affects the structure and clinical appearance of enamel of all or nearly all the teeth in a more or less equal manner and which may be associated with morphologic or biochemical changes elsewhere in the body [1].

The combination of AI and nephrocalcinosis may suggest a contiguous gene syndrome or pleiotropism. One hypothesis suggests that there is an underlying abnormality in the interstitial matrix, which leads to dystrophic calcification in the kidney and abnormal enamel production in the teeth. Involvement of two separate but closely linked genes has also been suggested [2]. Another hypothesis suggests that many of the dental proteins that were thought to be tissue-specific may also be traced in non-dental tissues and the role of these proteins in calcium and phosphate metabolism and renal function needs further research [3]. The genetic basis of AI and nephrocalcinosis syndrome is yet to be established.

The first report of this syndrome (occurring in a sibling pair) appeared in 1972 [4]. One sibling died at the age of 26 years, having suffered severe renal failure as a complication of nephrocalcinosis; the other developed multiple urinary infections, hypertension and renal failure. Subsequently reported cases share the following common features: failure of eruption, enamel agenesis, unexplained nephrocalcinosis with normal plasma calcium, alkaline phosphatase and parathyroid functions.

Here is a case report of siblings, children of consanguineous marriage with multiple retained hypoplastic deciduous teeth, showing unerupted permanent teeth with enlarged pericoronal follicle space, associated with nephrocalcinosis of both kidneys.

## Case presentation

A 13-year-old female patient along with her 18-year-old brother reported to the department with a complaint of gummy smile and discolored teeth. Both patients gave a history of delayed eruption of the permanent teeth. Medical history of both patients was non-contributory. The male patient had a history of extraction of maxillary deciduous incisors along with the surgical exposure of upper permanent incisors at the age of 12 years. In spite of the surgical exposure, teeth failed to erupt for the next 18 months. History revealed that the children are from a consanguineous family and neither of the parents had any developmental dental abnormality.

**Figure 1:**
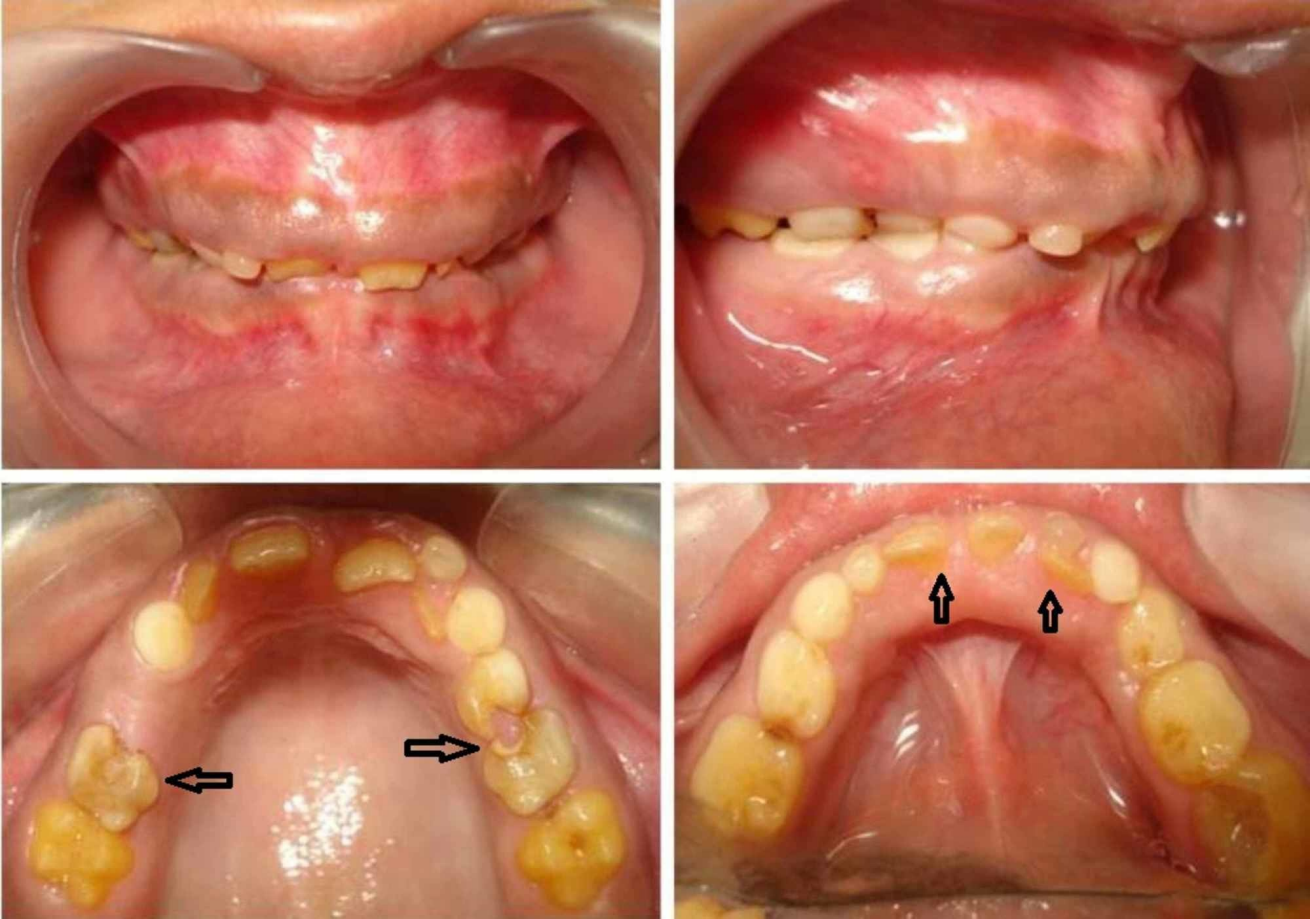
Intraoral view of the female patient

On extra oral examination, both patients presented with a convex profile, dolichocephalic head form and leptoprosopic facial form. Intraoral examination of the female patient showed multiple retained deciduous teeth which were severely attrited. There were only six permanent teeth present which include the maxillary central and left lateral incisors, mandibular central incisors (CI) and left the first molar. All the teeth present appeared to be hypoplastic (Figure 1).

**Figure 2:**
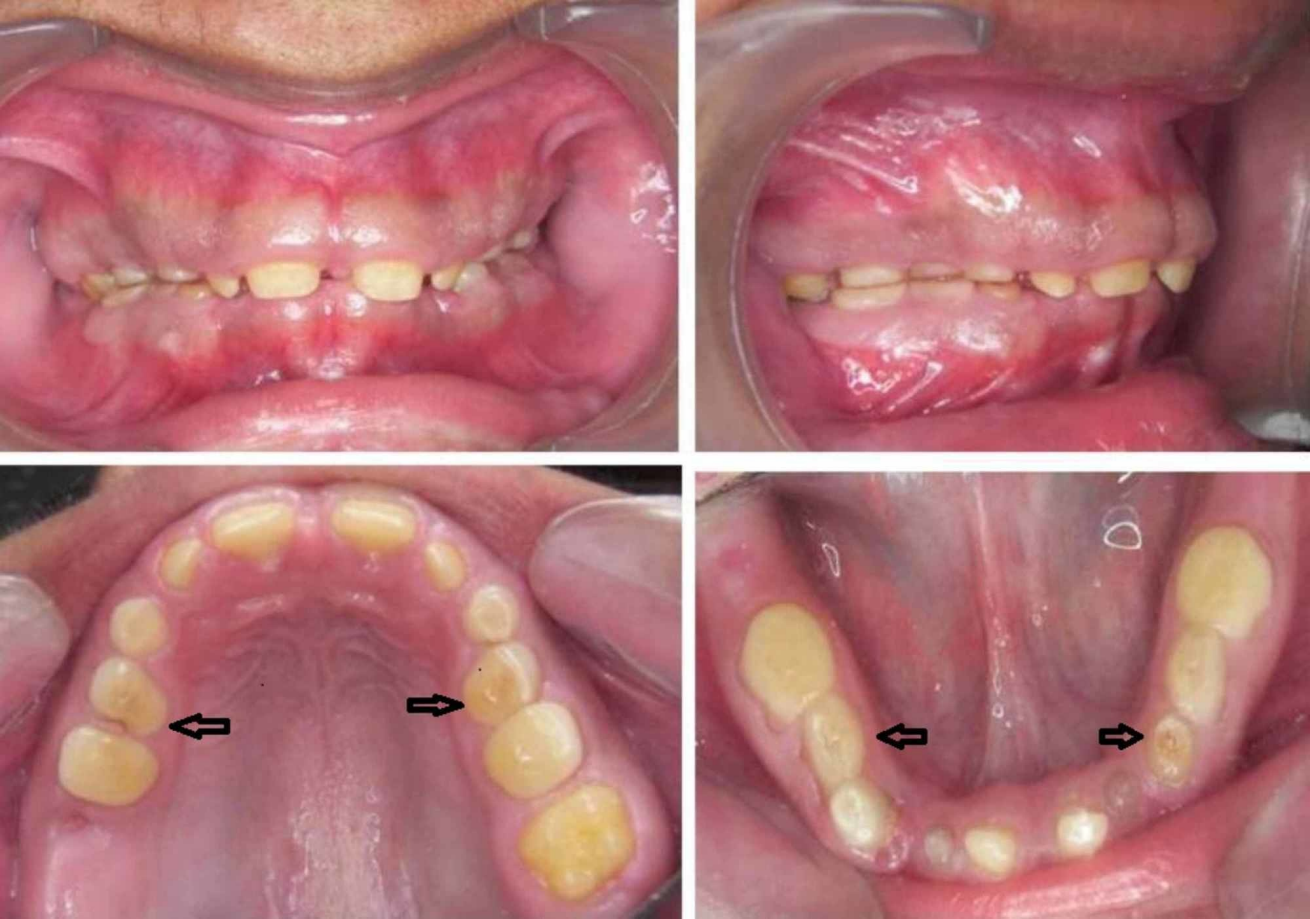
Intraoral view of the male patient

In contrast the male patient showed multiple retained deciduous teeth along with permanent maxillary central, lateral incisors and left first molar, mandibular CI. All the deciduous teeth were yellowish brown and severely attrited. The permanent teeth were hypoplastic and generalized microdontia was seen (Figure 2).

**Figure 3:**
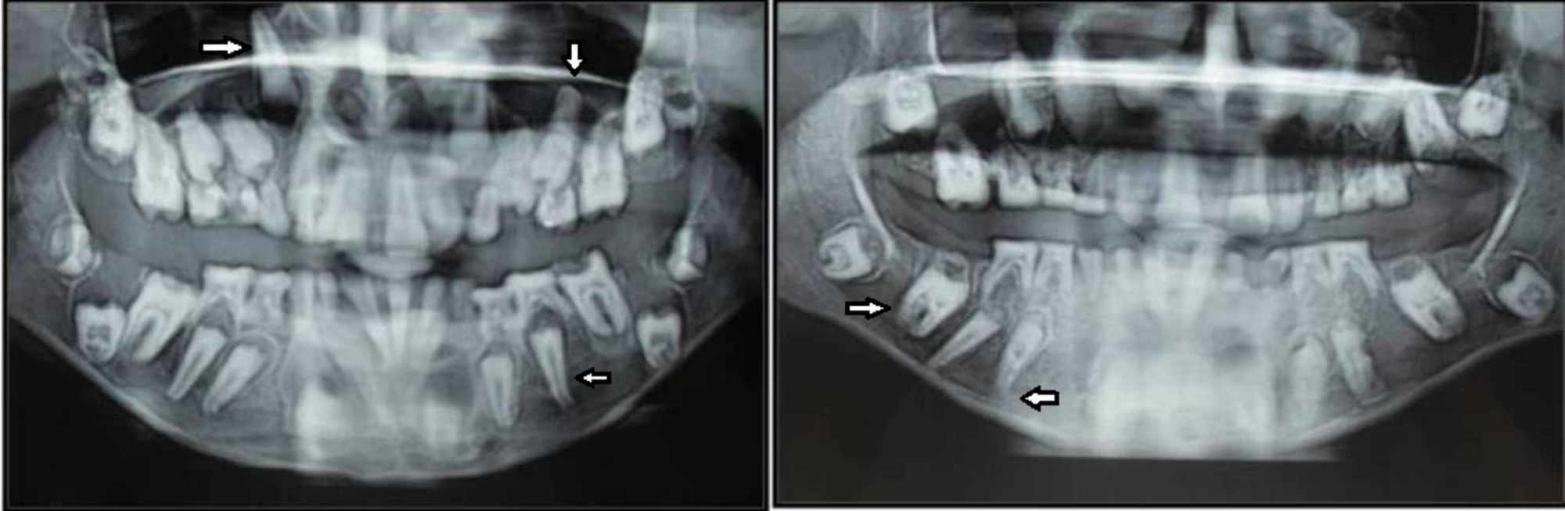
Orthopantamograph of the year 2009 of female and male patients shows apical displacement of tooth buds

Orthopantamograph (OPG) of both the patients revealed the presence of multiple unerupted permanent teeth. Enamel of all the teeth was hypoplastic along with flattening of crown (Figure 3).

**Figure 4:**
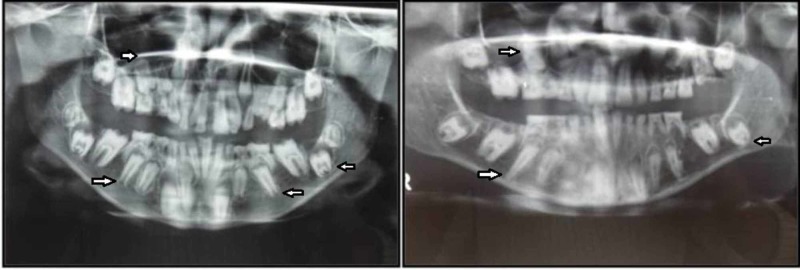
Orthopantamograph of the year 2007 of female and male patients shows multiple unerupted permanent teeth

The OPG was compared to the previous OPG which the patient was subjected to two years earlier (Figure 4).

In comparison with the previous OPG the present OPG showed the apical displacement of tooth buds along with the increase in the follicular space without hindering the formation of root in most of the teeth for both patients.

**Figure 5:**
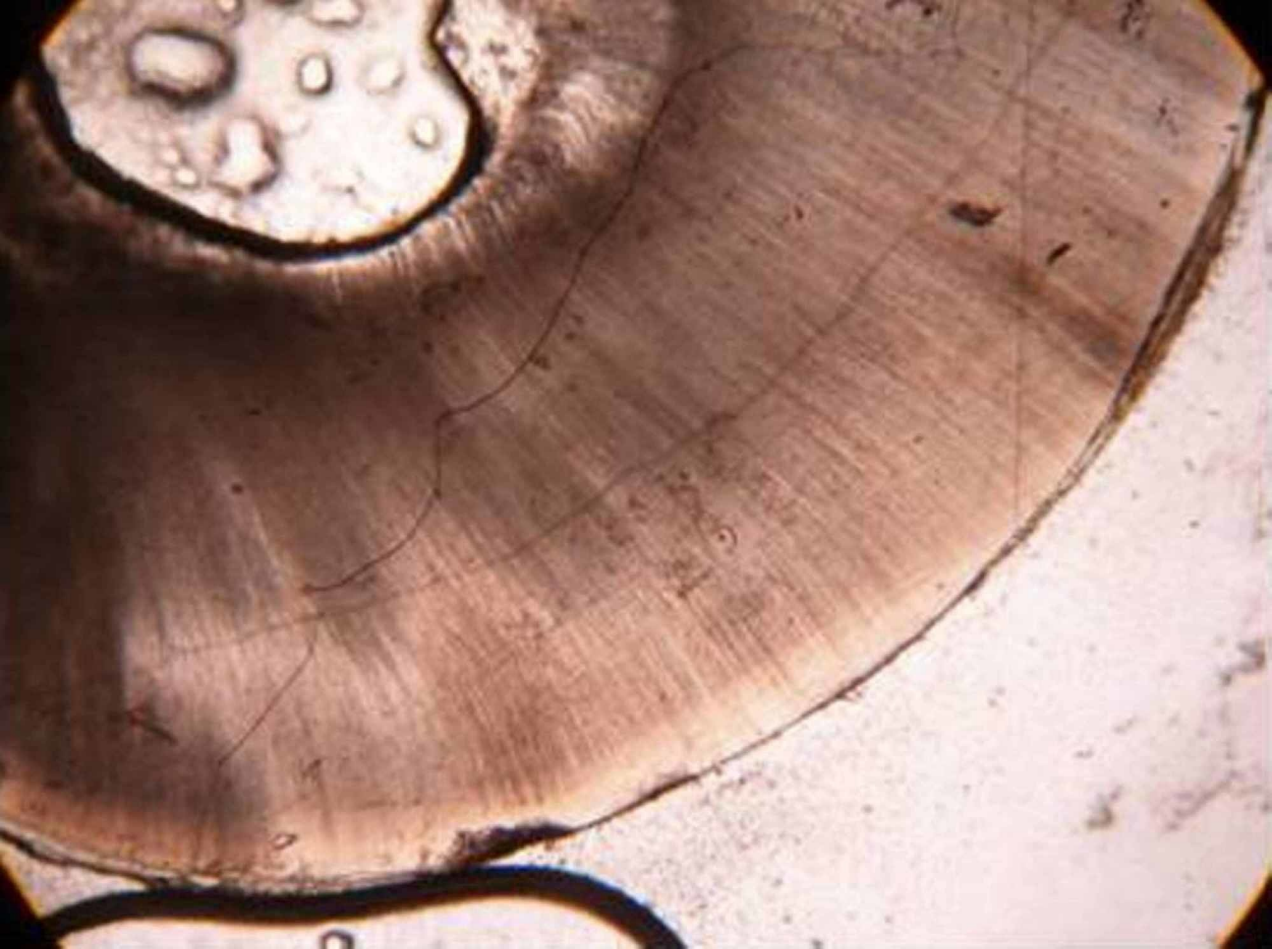
Histopathology picture of deciduous tooth specimen of female patient

A provisional diagnosis of AI, hypoplastic type was made, based on the history, clinical and radiographic presentation. An extracted deciduous tooth of the female patient was sent for histopathology, which confirmed presence of thin enamel with sparse and irregularly arranged enamel rods, with loss of enamel at incisal margins, with normal dentin architecture suggesting hypoplastic type of AI (Figure 5). Differential diagnosis includes enamel hypoplasia due to fluorosis and trauma.

**Figure 6:**
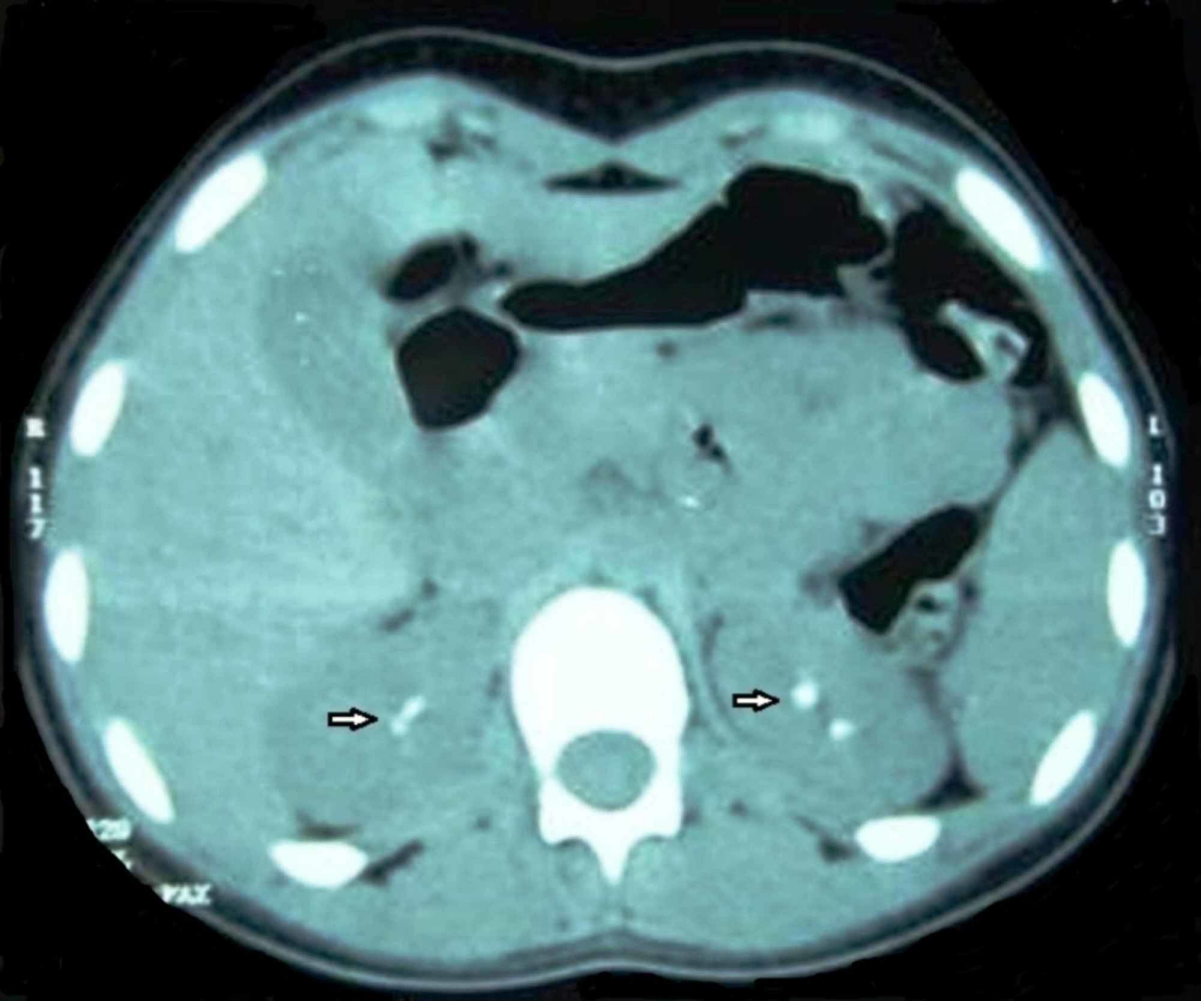
Axial CT scan of kidneys showing bilateral nephrocalcinosis (female patient)

Patients were also subjected to systemic investigations.The Investigations revealed the following results:

Ultrasonography (USG) report for the female patient revealed bilateral nephrocalcinosis of kidneys. Computed Tomography (CT) scan of upper abdomen also revealed calcifications in medulla and simple cortical cysts measuring 14x14 mm in lower pole of both kidneys and was diagnosed bilateral renal calculi, bilateral nephrocalcinosis (Figure 6).

**Figure 7:**
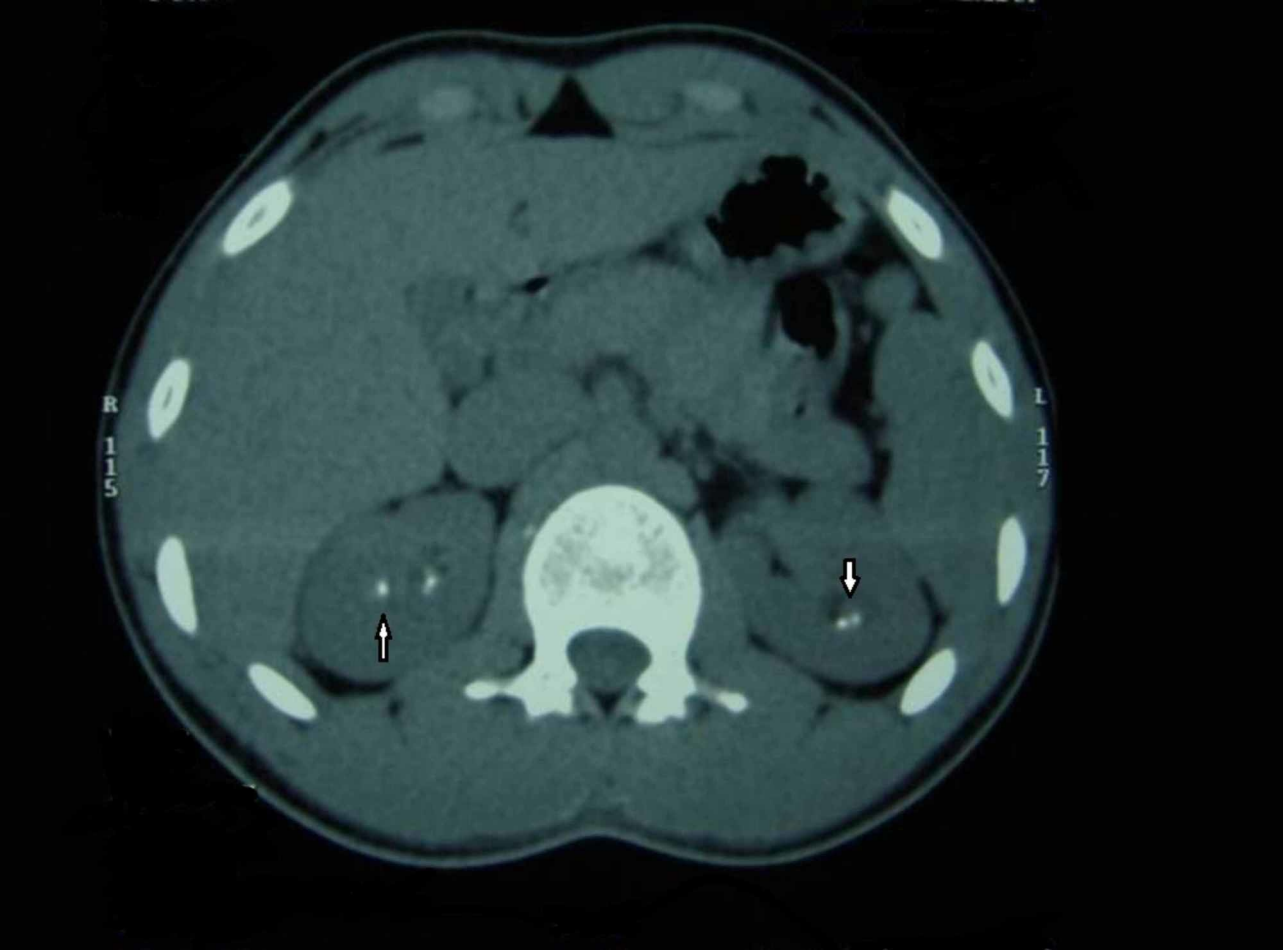
Axial CT scan of kidneys showing bilateral nephrocalcinosis (male patient)

CT scan of upper abdomen for the male patient revealed bilateral multiple renal calculi largest measuring approx 6mm, screening USG show specks of cortical and medullary calcification and was diagnosed as bilateral nephrocalcinosis (Figure 7).

Thyroid and parathyroid profile blood assays were normal for both patients but there was an increase in alkaline phosphatase levels 403.00 IU/L (50-162 IU/L) for the female patient which may be considered normal for her age (children 70-570 IU/L).

**Figure 8:**
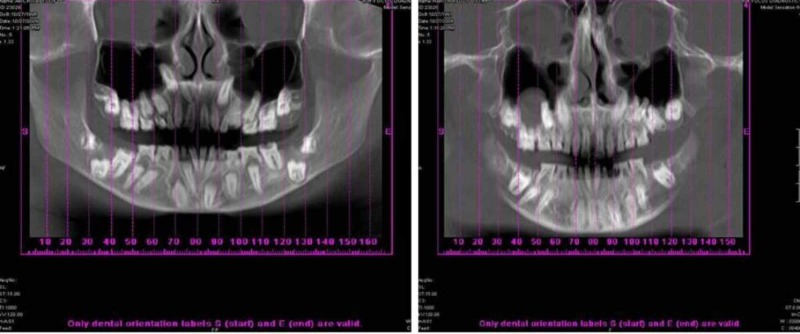
CT of maxilla and mandible of female and male patient

Dental CT findings correlated with the findings of the OPG and there was no evident bone pathology relating to the non eruption of the teeth (Figure 8).

**Figure 9:**
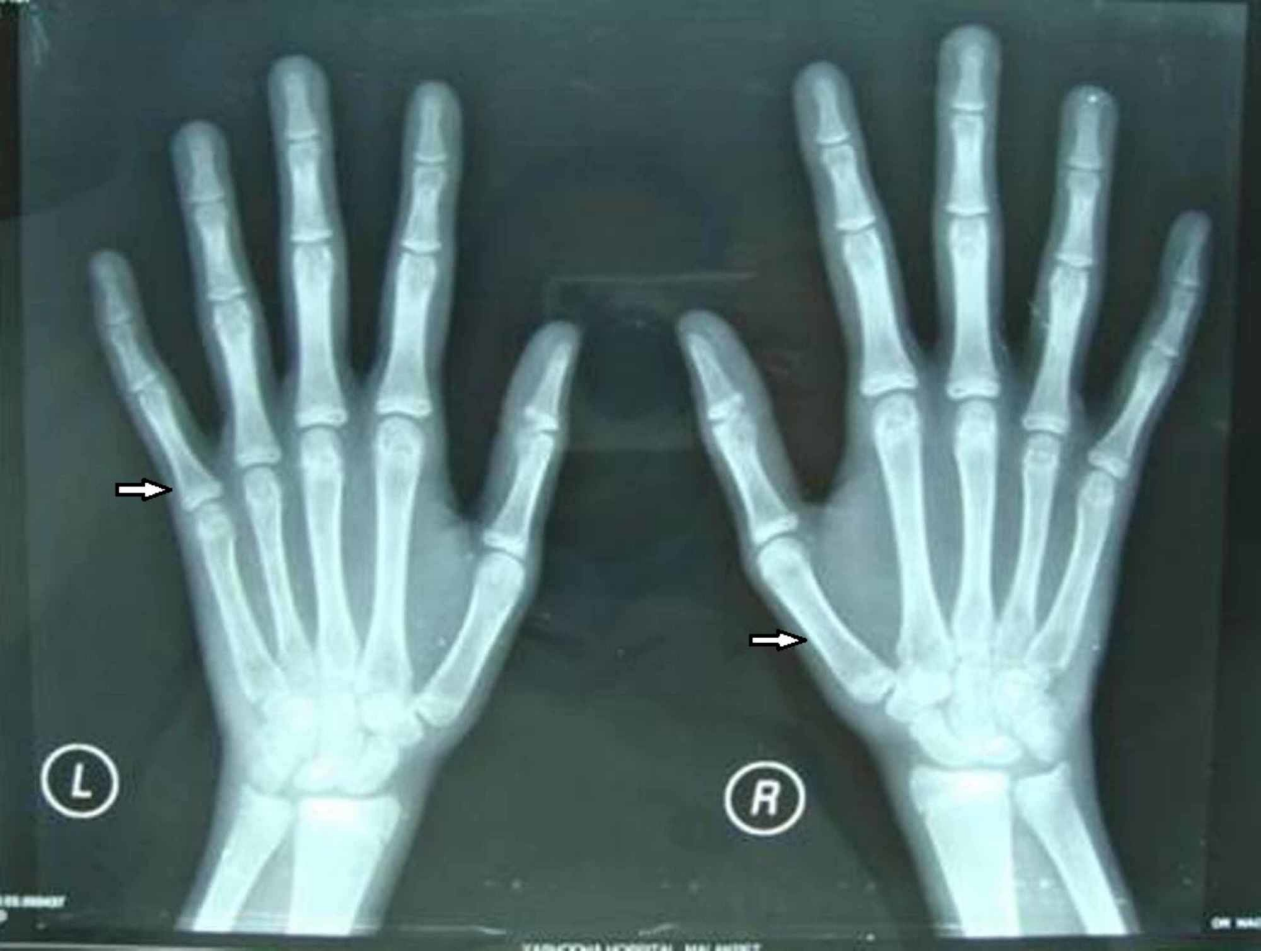
Hand-wrist X-ray of female patient

A hand-wrist X-ray was advised which showed diffuse osteopenia (Figure 9).

Since both the patients were diagnosed with AI and nephrocalcinosis, they were referred to urologist. All the test results were within normal limits for both the patients.

## Discussion

AI shows autosomal dominant, autosomal recessive, sex-linked and sporadic inheritance patterns [1]. It mainly affects the quality and/or quantity of dental enamel. AI is also associated with multiorgan syndromes like nephrocalcinosis, platyspondyly, hypothalamo-hypophyseal insufficiency, Kohlschutter syndrome and cone-rod dystrophy. Unerupted teeth, pulpal calcifications, inter radicular dentinal dysplasia, root and crown resorption, cementum deposition, truncated roots, anterior open bite, and taurodontism coexists with amelogenesis imperfecta [5].

AI can be differentiated into three groups: hypoplastic, hypocalcified, hymomature, and there are fourteen different hereditary subtypes of AI [6]. The present cases can be diagnosed as generalized thin hypoplastic AI.

To date, mutations in five genes (AMELX, ENAM, DLX3 KLK4 and MMP-20) have been found to cause AI. AMELX is responsible for X-linked AI, ENAM and DLX-3 are autosomal dominant, and KLK4 and MMP-20 are autosomal recessive (AR). Many of the dental proteins that were believed to be tissue-specific have been traced in the kidney, for example, DLX3, a homeobox protein, is also present in the kidney [7].

Previous studies describe families with an AR mode of inheritance and generalized thin phenotype (enamel) that in some instances show failure of tooth eruption [8]. This could be explained by the pathology of the dental follicles which might be unable to synthesize the factors that initiate eruption. It could also obstruct the eruption by mechanical retention due to cystic or fibrous transformation [9].

Comparison of the panoramic radiograph taken at presentation with the film taken approximately two years earlier revealed that, there had been little or no axial movement of teeth through bone in the intervening period, this could happen because of growth of the alveolar process proper, failure of eruption potential of the permanent teeth or pericoronal cystic activity or accumulation of fluid in perifollicular space can also be suspected. The latter is true particularly in relation to all permanent canines and mandibular second molars in the female patient.

On the contrary, there was bone of normal density covering the occlusal surfaces of the mandibular first permanent molar teeth; there is generalized passive eruption of the deciduous teeth this resulting in unimpeded alveolar growth thus resulting in an excess lower anterior facial height, the reason of the gummy smile could be attributed to it.

From a dental viewpoint, the management of patients presenting with primary failure of eruption is extremely difficult, since the teeth involved tend to ankylose when orthodontic traction is applied. In the absence of a normal response to orthodontic force, the only way to move unerupted teeth into occlusion is to reposition them surgically without disturbing the periodontal ligament. When this is not feasible, prosthetic replacement of the abnormal teeth becomes the only treatment possible. In the present case, a combined surgical and orthodontic approach is being considered to bring the unerupted teeth into alignment.

All children with AI and their parents should be questioned regarding existing or past medical problems with particular reference to urinary tract. Urine examination for infection or proteinuria should be done and the child be referred for medical examination including renal function and ultrasonography to detect nephrocalcinosis [2].

## Conclusions

Awareness of potential association of AI with nephrocalcinosis is essential among dentists as they can early refer affected individuals to nephrology services and hence has improved prognosis. Knowledge of occurrence of this syndrome in consanguineous and non consanguineous families, proper medical history and systemic examination, including renal ultrasound and renal function tests in patients with AI helps in early diagnosis and reduces the morbidity associated with unrecognized renal disease.
